# Antibiotic Susceptibility Patterns of Bacterial Isolates from Pus Samples in a Tertiary Care Hospital of Punjab, India

**DOI:** 10.1155/2016/9302692

**Published:** 2016-10-30

**Authors:** Rugira Trojan, Lovely Razdan, Nasib Singh

**Affiliations:** ^1^Department of Paramedical Sciences, Lovely Professional University, Punjab, India; ^2^Department of Pathology, Patel Hospital, Jalandhar, Punjab, India; ^3^Department of Microbiology, Eternal University, Baru Sahib, Himachal Pradesh, India

## Abstract

We determined the prevalence and antibiotic susceptibilities patterns of bacterial isolates from pus samples collected from patients in a tertiary care hospital of Punjab, India.* E. coli* was the most prevalent pathogen (51.2%) followed by* Staphylococcus aureus* (21%),* Klebsiella pneumoniae* (11.6%),* Pseudomonas aeruginosa* (5.8%),* Citrobacte*r spp. (3.5%),* Acinetobacter baumannii* (2.3%),* Proteus mirabilis* (2.3%), and* Streptococcus* spp. (2.3%).* E. coli*,* K. pneumoniae*,* A. baumannii*, and* Citrobacter* isolates were resistant to multiple antibiotics including higher generation cephalosporins.* S. aureus* and* Streptococcus* isolates were sensitive to cloxacillin and vancomycin. However,* P. aeruginosa*,* P. mirabilis*, and* Streptococcus* isolates were found to be less resistant to the spectrum of antibiotics tested. Overall, our findings indicate the prevalence of resistance to different classes of antibiotics in bacterial isolates from pus infections and hence highlight the need for effective surveillance, regulator reporting, and antibiogram-guided antibiotic prescription.

## 1. Introduction

The human skin and soft tissue infections (SSTIs) caused by microbial pathogens during or after trauma, burn injuries, and surgical procedures result in the production of pus, a white to yellow fluid comprised of dead WBCs, cellular debris, and necrotic tissues [1–3]. Both aerobic and anaerobic bacteria have been implicated in wound infections which commonly occur under hospital environment and result in significant morbidity, prolonged hospitalization, and huge economic burden [4]. The emergence antibiotic resistance and its rapid spread of among pathogenic bacterial isolates are considered as grave threats to the public health worldwide. During the last few decades, multidrug-resistant Gram-negative bacterial strains such as* Acinetobacter baumannii*,* E. coli*,* Klebsiella pneumoniae*,* Pseudomonas aeruginosa,* and Gram-positive methicillin-resistant* Staphylococcus aureus* (MRSA) were increasingly associated with pus infections under hospital settings due to extensive misprescription and inadequate dose regimen of antibiotics [5–7]. Rapid emergence of multidrug-resistant bacteria poses a serious threat to public health globally due to the limited treatment options and lukewarm discovery of new classes of antibiotics [7, 8]. The objective of this study is to characterize the pyogenic bacteria from pus samples and to determine their antibiotic susceptibilities to various generations of antibiotics commonly used in chemotherapeutic interventions.

## 2. Materials and Methods

### 2.1. Sample Collection and Characterization

A total of 143 pus samples were collected by sterile syringe aspiration (*n* = 41) and by sterile swabs (*n* = 102) from inpatients and outpatients of different wards of Patel Hospital, Jalandhar, Punjab (India), over a period of 5 months from January 2014 to May 2014 in accordance with standard protocols and ethical guidelines. Pus samples were collected from skin (furuncles, pustules, and abrasions), nasal wounds, ears, legs, internal organs (lungs, kidney, and bladder), and catheters. Pus samples were kept in Cary-Blair transport medium until processed for Gram staining and culturing. The samples were aseptically inoculated on blood agar (with 5% sheep blood) and MacConkey agar plates, incubated aerobically at 35°C–37°C for 24–48 h. Identification and characterization of isolates were performed on the basis of Gram staining, microscopic characteristics, colony characteristic, and biochemical tests using standard microbiological methods.

### 2.2. Antimicrobial Agents

Antibiotics discs containing amikacin (30 *μ*g), amoxicillin-clavulanic acid (30 *μ*g), aztreonam (30 *μ*g), ampicillin (10 *μ*g), azithromycin (30 *μ*g), cefepime (30 *μ*g), Cefoperazone/Sulbactam (75/30 *μ*g), ceftriaxone (30 *μ*g), cefotaxime (30 *μ*g), cefuroxime (30 *μ*g), cephalexin (30 *μ*g), ciprofloxacin (1 *μ*g), clindamycin (2 *μ*g), cloxacillin (30 *μ*g), trimethoprim/sulfamethoxazole (25 *μ*g), ertapenem (10 *μ*g), erythromycin (15 *μ*g), gatifloxacin (5 *μ*g), gentamicin (10 *μ*g), imipenem (10 *μ*g), levofloxacin (5 *μ*g), linezolid (30 *μ*g), meropenem (10 *μ*g), netilmicin (30 *μ*g), norfloxacin (10 *μ*g), ofloxacin (5 *μ*g), piperacillin-tazobactam (100/10 *μ*g), teicoplanin (30 *μ*g), tetracycline (30 *μ*g), and vancomycin (30 *μ*g) were obtained from Himedia Laboratories (Mumbai, India) and used as per manufacturer's instructions.

### 2.3. Antibiotics Susceptibility Testing

Antibiotic susceptibilities of bacterial isolates were determined according to the method recommended by the Clinical and Laboratory Standards Institute [9]. Briefly, inocula were prepared for each bacterial isolate by adjusting the turbidity to 0.5 McFarland standard and spread on Muller-Hinton agar plates. Antibiotic discs (Himedia, Mumbai, India) were placed on the agar plates and incubated overnight at 37°C for 24 h. The zones of inhibition were measured and the isolates were classified as sensitive, intermediate, and resistant according to CLSI tables and guidelines [9].

## 3. Results and Discussion

**Figure 1:**
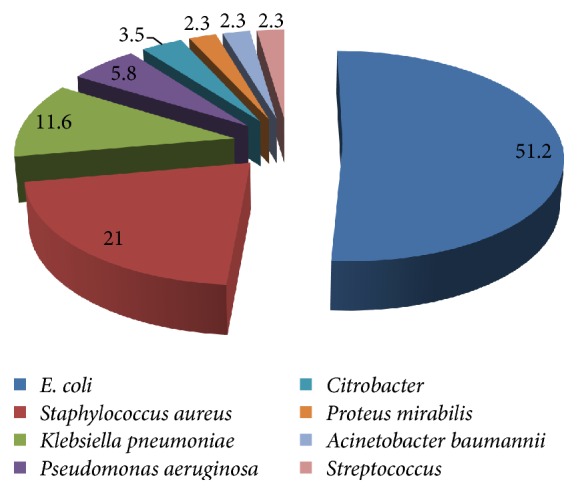
Distribution of bacterial pathogens (%) isolated from pus samples at a tertiary care hospital in Punjab, India.

Of the 143 pus samples collected from different wards of the hospital, 86 samples (60.1%) showed bacterial growth after 24–48 h of incubation whereas 57 samples (39.9%) were negative for growth. Based on Gram staining, morphological features, culture characteristics, and biochemical characterization, the bacterial isolates were assigned to eight bacterial species.* E. coli* was the most frequent pathogen as revealed by 51.2% occurrence followed by* S. aureus* (21%),* K. pneumoniae* (11.6%),* P. aeruginosa* (5.8%), and* Citrobacte*r spp. (3.5%) and approximately 2.3% each was represented by* A. baumannii*,* P. mirabilis*, and* Streptococcus* spp. (Figure 1). Gram-negative bacteria were the dominant isolates (77%) from pus samples compared to Gram-positive bacteria which are in agreement to several earlier studies. Our findings correlate with Zhang et al. [10] who reported predominance of* E. coli*,* S. aureus*,* K. pneumoniae*, and* P. aeruginosa* in pus samples from patients with severe intra-abdominal infection. In another study,* S. aureus* was the dominant bacterial species from wounds followed by* P. aeruginosa*,* P. mirabilis*,* E. coli*, and* Corynebacterium* spp. [11]. According to Dryden [12],* S. aureus* and MRSA are major cause of soft tissue infections in hospitalized patients. Several other reports have also implicated* Pseudomonas*,* Staphylococcus*,* Streptococcus*,* Klebsiella*, and* E. coli* in wound infections [6, 13]. Antibiogram results from the present study show that* E. coli* was more resistant to amoxicillin-clavulanic acid, cephalosporins, while being least resistant to amikacin, imipenem, gentamicin, and meropenem (Table 1). On the other hand,* A. baumannii* showed extensive multidrug resistance pattern as it was resistant to all the antibiotics.* P. aeruginosa* was more susceptible to tested antibiotics compared to* K. pneumoniae*. Both species showed resistance to cephalosporins. Previous studies from Canada, Croatia, and Latin America found* P. aeruginosa* isolates resistant to carbapenems, aminoglycosides, and ciprofloxacin but not to piperacillin [14, 15].* Citrobacter* isolates showed resistance to eight antibiotics whereas they were moderately susceptible to other antibiotics.* S. aureus* was highly susceptible to vancomycin (100%), linezolid (100%), imipenem (89%), and meropenem (84%) while it showed resistance to ampicillin, amoxicillin-clavulanic acid, ciprofloxacin, and azithromycin. Unlike some reports in which MRSA was associated with wound infections [16, 17], our findings revealed susceptibility in* S. aureus* isolates towards cloxacillin and cephalosporins.* P. mirabilis* and* Streptococcus* isolates, however, exhibited minimal resistance and were susceptible to most of the antibiotics (Table 1). Both Gram-positive isolates were fully susceptible to vancomycin and linezolid.

This study provides the evidence of high prevalence of antibiotic resistant bacteria in pus samples of patients collected from a tertiary care hospital environment. Our findings indicate the predominance of* E. coli* among the bacterial isolates of pus. The prevalence and antibiotics resistance patterns of pyogenic bacterial isolates usually exhibit variability according to geographic areas and climate conditions. Existence of high drug resistance to multiple antibiotics in* E. coli*,* S. aureus*,* K. pneumoniae*, and* P. aeruginosa* isolates from pus samples in this study and several other related reports points towards negligence on patients part, incomplete treatment schedules, antibiotics misuse, self-prescription, misprescription, lack of regional antibiogram data, and limited knowledge about multidrug-resistant isolates and antimicrobial resistance among clinicians. Updated knowledge of antimicrobial susceptibility profiles of clinical isolates will not only assist in designing the most appropriate dose-regimen and treatment schedule against wound infections but also help in curbing the alarmingly expanding menace of drug resistance.

## 4. Conclusion

In conclusion, pyogenic wound infections were found prevalent in the tertiary care hospital and* E. coli* isolates showed highest incidence followed by* S. aureus*,* P. aeruginosa*,* K. pneumoniae*,* A. baumannii*,* Citrobacter*,* P. mirabilis*, and* Streptococcus* spp. Bacterial isolates exhibited high to moderate levels of resistance against different classes of antibiotics. The susceptibility data from this report may be worth consideration while implementing empiric treatment strategies for pyogenic infections. At the same time, strict health policies should also be implemented to regulate the purchase and prescription and restrict the unsupervised antibiotic use as well as continuous monitoring and reporting antibiotic resistance.

## Figures and Tables

**Table 1:** Antibiotic susceptibilities of Gram-negative and Gram-positive bacteria isolated from pus samples at a tertiary care hospital in Punjab, India.

Antibiotics	Antibiotic sensitivity (%) of bacterial isolates							
	*E. coli* (*n* = 44)	*Pseudomonas aeruginosa* (*n* = 5)	*Klebsiella pneumoniae* (*n* = 10)	*Proteus mirabilis* (*n* = 2)	*Citrobacter *spp. (*n* = 3)	*Acinetobacter baumannii* (*n* = 2)	*Staphylococcus aureus* (*n* = 18)	*Streptococcus *spp. (*n* = 2)
Amikacin	75	80	50	100	0	0	nt	nt
Amoxicillin-clavulanic acid	5	0	0	50	0	0	11	50
Aztreonam	19	40	10	50	0	0	nt	nt
Ampicillin	nt	nt	nt	nt	nt	nt	11	50
Azithromycin	nt	nt	nt	nt	nt	nt	23	100
Cefepime	16	20	10	50	33	0	34	50
Cefoperazone/Sulbactam	34	20	10	100	33	0	nt	nt
Ceftriaxone	30	20	10	50	33	0	67	100
Cefotaxime	21	0	20	50	0	0	45	100
Cefuroxime	5	0	10	0	0	0	78	100
Cephalexin	nt	nt	nt	nt	nt	nt	67	100
Ciprofloxacin	21	60	40	100	67	0	11	100
Clindamycin	nt	nt	nt	nt	nt	nt	73	100
Cloxacillin	nt	nt	nt	nt	nt	nt	100	100
Trimethoprim/sulfamethoxazole	32	20	20	50	0	0	34	50
Ertapenem	27	nt	30	50	33	0	nt	nt
Erythromycin	nt	nt	nt	nt	nt	nt	28	100
Gatifloxacin	21	40	40	50	66	0	nt	nt
Gentamicin	46	60	40	50	0	0	nt	nt
Imipenem	75	80	50	50	67	0	89	100
Levofloxacin	25	60	30	50	67	0	28	100
Linezolid	nt	nt	nt	nt	nt	nt	100	100
Meropenem	68	80	40	50	67	0	84	100
Netilmicin	57	80	30	50	33	0	nt	nt
Norfloxacin	14	20	20	50	67	0	nt	nt
Ofloxacin	16	40	30	100	67	0	nt	nt
Piperacillin-tazobactam	36	80	40	100	33	0	28	100
Teicoplanin	nt	nt	nt	nt	nt	nt	78	100
Tetracycline	nt	nt	nt	nt	nt	nt	50	50
Vancomycin	nt	nt	nt	nt	nt	nt	100	100

nt: not tested.
